# Can Recovery From an Eating Disorder Be Measured? Toward a Standardized Questionnaire

**DOI:** 10.3389/fpsyg.2018.02456

**Published:** 2018-12-11

**Authors:** Rachel Bachner-Melman, Lilac Lev-Ari, Ada H. Zohar, Shay Lee Lev

**Affiliations:** ^1^Clinical Psychology Graduate Program, Ruppin Academic Center, Hadera, Israel; ^2^The Paul Baerwald School of Social Work and Social Welfare, The Hebrew University of Jerusalem, Jerusalem, Israel; ^3^Student Counselling Service, University of Haifa, Haifa, Israel

**Keywords:** eating disorders, recovery, questionnaire, family members, exploratory factor analysis, confirmatory factor analysis, eating disorder clinicians

## Abstract

**Background:** There is a clear need for a standardized definition of recovery from eating disorders (EDs) and for self-report instruments to assess where individuals with an ED are situated at a given point of time along their process of illness and recovery. It has been acknowledged that psychological and cognitive symptoms are important to recovery in addition to physical and behavioral indices. This study proposes a 28-item multidimensional questionnaire encompassing the main features of recovery from ED, derived from the endorsement of different criteria by people with a lifetime ED diagnosis, family members and ED clinicians.

**Methods:** Participants were 213 volunteers over the age of 18 (118 people with a lifetime ED diagnosis, 58 healthy family members of people with EDs and 37 ED clinicians), who completed the ED-15 and indicated online how important they thought each of 56 criteria were for recovery from an ED.

**Results:** Four factors were identified in an exploratory factor analysis: Lack of Symptomatic Behavior (LSB), Acceptance of Self and Body (ASB), Social and Emotional Connection (SEC), and Physical Health (PH). Confirmatory factor analysis using the seven highest loading items from each subscale confirmed the structure validity of a shortened version of this questionnaire, the Eating Disorders Recovery Endorsement Questionnaire (EDREQ), which had excellent goodness-of-fit indices. Despite a few between-group differences, there was general agreement that LSB was most salient to recovery, followed by ASB, SEC, and PH in that order.

**Conclusion:** Despite the absence of a standardized definition of recovery from ED, there is a general consensus about its components. The EDREQ is a psychometrically sound questionnaire containing items that people with an ED history, their family members and therapists all define as important components of recovery. The inclusion of emotional and psychosocial aspects of recovery in addition to symptomatic and medical aspects is important to expand treatment goals and the concept of recovery from EDs beyond symptom relief and the absence of disease markers. As a clinical tool, the EDREQ stands to assist in setting and refining therapeutic goals throughout therapy, and in establishing standardized, comparable norms for recovery levels in research.

## Introduction

Eating disorders (EDs) are psychiatric disorders characterized by eating disturbances that significantly impair physical and psychological health, and have a poor prognosis (Steinhausen, 2009). Although many people who develop EDs do recover partially or completely, estimations of recovery rates from ED vary hugely (Couturier and Lock, 2006; Berkman et al., 2007). This lack of clarity is due, to a large extent, to differing definitions and methods of measuring recovery (Jarman and Walsh, 1999; Bachner-Melman et al., 2006; Berkman et al., 2007; Bardone-Cone et al., 2010; McGilley and Szablewski, 2010; Noordenbos, 2011). The DSM-5 defines illness and remission, but not recovery (American Psychiatric Association, 2013). There is a clear need for a standardized definition of recovery from EDs (Dawson et al., 2015; Bardone-Cone et al., 2018) and for self-report instruments to assess where specific individuals diagnosed in the past with an ED are situated at a given point of time along their process of illness and recovery.

In most studies on ED outcome or recovery, recovery has been defined using the medical model that includes various combinations of physical and behavioral/symptomatic criteria. Some studies focusing on recovery from anorexia nervosa (AN) used the criterion of body mass index (BMI) alone. For example, Walsh et al. (2006) used a cutoff of 19, and Carter et al. (2004, 2012) used a cutoff of 20. Others used a combination of body weight and menstruation (Fichter and Quadflieg, 1999; Lowe et al., 2001; Eisler et al., 2007), which forms the basis of the “Morgan-Russell” criteria that define AN outcome as “good,” “intermediate,” “poor,” or “died” (Morgan and Russell, 1975). In his systematic review of 119 studies on the outcome of AN, Steinhausen (2002) reported that in these studies, recovery was defined, in general, as remission from essential clinical symptoms.

This is true also for studies on outcome or recovery from EDs other than AN, which tend to frame recovery around clinically relevant symptomatic change. The Psychiatric Status Rating (PSR) scale (Herzog et al., 1993), a 6-point, symptom-based scale assessing the level of AN and bulimia nervosa (BN) symptomatology according to the DSM-IV, has been used to define recovery in some studies (Kordy et al., 2002; Bloks et al., 2004), including at least one on the “outcome” of binge eating disorder (BED; Fichter et al., 2008). Over 80% of the 126 studies included in Vall and Wade (2015) systematic review of predictors of ED outcomes defined outcomes on the basis of symptom remission. Suggested criteria for recovery from BN have included, in addition to the remission of behavioral symptoms, the undue influence of weight and shape on self-evaluation (Cogley and Keel, 2003), the absence of substance abuse or dependence and the absence of psychoactive medication such as antidepressants (Kaye et al., 1998; Bailer et al., 2004). Recovery from BED has been studied less than from AN and BN (see for example Fairburn et al., 2000; Krentz et al., 2007). A review of the criteria adopted, while beyond the scope of this paper, would be a welcome addition to the literature. Since there are differences between the course and outcome of EDs in adolescents and adults (Fisher, 2003), it seems surprising that differences between these two populations in criteria for recovery do not seem to have been explored. Although symptomatic improvement is an integral part of recovery, it seems misleading to define recovery using this yardstick, since symptomatic improvement, in particular weight gain in the case of AN, is by definition accompanied by internal distress in the short term. It would therefore seem more accurate to define biological or medical recovery as a precondition for, rather than a definition of recovery.

Some, but not all studies stipulate a minimal time period for symptom-based recovery criteria, which also varies between studies. Strober et al. (1997) and Pike (1998), for example, defined recovery as meeting remission criteria [≥90% of ideal body weight, regular menstruation, absence of compensatory behaviors, and Eating Disorder Examination subscales within 2 standard deviations (SD) of normal] for at least 8 weeks, and Bachner-Melman et al. (2006) included the absence of binging and purging symptoms for at least 8 consecutive weeks. Levallius et al. (2015) defined recovery as a lack of DSM-IV symptoms in the past 90 days, and Bardone-Cone et al. (2010) included in her stringent criteria for full recovery the absence of binge eating, purging and fasting in the past 3 months. The criteria for recovery in studies by Kordy et al. (2002) and by Zerwas et al. (2013) had to be met for at least 1 year.

Some empirical studies have included measures that go beyond biological and behavioral DSM symptoms. Martin (1985), for example, included a five-point scale assessing “social and family life” in her global rating scale of 25 adolescents treated for AN. Weight and shape concerns as assessed by the Eating Disorders Examination-Questionnaire (EDE-Q) have been included in the definitions adopted by some researchers, for example Bardone-Cone et al. (2010) required all EDE-Q subscales to be within one standard deviation of population norms, as was recommended also by Khalsa et al. (2017). Other studies defined recovery from ED as including a lack of body dissatisfaction (Eckert et al., 1995; Bachner-Melman et al., 2006; Lindner et al., 2014), a lack of fear of weight gain and of body image distortion (Bachner-Melman et al., 2006; Lindner et al., 2014) and self-esteem that is not dependent on body and shape (Lindner et al., 2014).

A distinction between criteria for full versus partial recovery has not been consistently proposed in the literature. A European collaboration of experts (COST Action B6) suggested criteria for partial remission from restricting AN (BMI > 17.5, no binging or purging for 1 month), binge-purge AN (BMI > 17.5, no purging, ≤1 binge per week for 1 month) and BN (≤1 binge/purge attacks per week) and criteria for full remission from both subtypes of AN (BMI > 19, no binging or purging, no extreme fear of weight gain for 3 months) and from BN (no binging or purging, no extreme preoccupation with figure for 3 months). Recovery was defined using the same criteria for full recovery for a duration of 1 year. Khalsa et al. (2017) proposed including EDE scores within one standard deviation of population norms as a criterion for full recovery and within 1.5 standard deviations of population norms as a criterion for partial recovery. Cogley and Keel (2003) included in the criteria for full as opposed to partial recovery from BN scores of 3 or lower on the Weight and Shape Concern subscales of the Eating Disorders Examination (EDE; Cooper and Fairburn, 1987). Cognitive recovery from BN in addition to behavioral recovery therefore distinguished full recovery from partial recovery, as in the study by Bachner-Melman et al. (2006) on AN. Other studies also make this distinction between full and partial recovery, but it is no less arbitrary than criteria for recovery in general.

Definitions of recovery in empirical studies described above are not only variable and arbitrary, but they are limited by having been determined by medical professionals and researchers, but not by people with personal experience of EDs. Another major drawback of studies that measure recovery predominantly in terms of symptom reduction is that they omit the dimensions of growth and the development of wellness from the concept of recovery (Bardone-Cone et al., 2018). A growing body of qualitative research on recovery from EDs has clearly shown that from the point of view of people with lived experience of EDs, symptom change only partially captures the essence of recovery from ED and additional factors are central to the process (Lamoureux and Bottorff, 2005; Björk and Ahlstrom, 2008; Jenkins and Ogden, 2011; Emanuelli et al., 2012; Linville et al., 2012; Hay and Cho, 2013; Bardone-Cone et al., 2018). This literature has added a wealth of psychological dimensions to the concept of recovery, many of them non-specific to EDs, such as quality of life and psychological, social and emotional functioning. These dimensions tap into the concept of well-being that has received much research attention in the field of positive psychology in recent decades (Keyes et al., 2002; de Vos et al., 2017).

Patching and Lawler (2009) found that as the women interviewed in their study recovered, they re-engaged with life, developed conflict resolution skills and rediscovered their sense of self. This sense of identity, authenticity and empowerment was also a central finding in studies by Lamoureux and Bottorff (2005); Jenkins and Ogden (2011), Bowlby et al. (2012), and Duncan et al. (2015). Lindgren et al. (2015) found that self-acceptance in a broad sense was an important part of recovery from BN. Espindola and Blay (2009) and Hay and Cho (2013) highlighted positive life experiences and satisfying interpersonal relationships as components of recovery. Bezance and Holliday (2013) similarly emphasized, for adolescents, how central improving relationships with peers and family members is to recovery from an ED.

The view of recovery as an ongoing and highly individual experience, rather than a biomedical disease, is a recent and in many ways a new concept in the mental health field (White et al., 2005). The “recovery model” (Anthony, 1993), which has been applied to EDs (Dawson et al., 2014), embraces a holistic, person-centered approach to recovery that emphasizes personal attitudes, values, feelings, goals, and strengths, alongside limitations caused by illness. Paradoxically, this model therefore leaves room for residual and ongoing symptoms, while defining recovery as “the development of new meaning and purpose in one’s life as one grows beyond the catastrophic effects of mental illness” (Anthony, 1993, p. 527).

The medical model and recovery model have been viewed as conflicting, and there certainly appears to be a tension between the notion of “full recovery” that includes complete symptom remission (Bachner-Melman et al., 2006; Bardone-Cone et al., 2010) and the notion that recovery can occur despite active symptoms. However, it has been claimed that these paradigms are complementary rather than conflicting and that the recovery model can be seen as an extension of the medical model (Barber, 2012; Flaherty, 2012). Indeed, whereas some individuals recovered from ED perceive recovery as coping with residual impulses to engage in symptomatic behavior, others experience themselves as having completely overcome such impulses (Björk et al., 2012). It is not quite clear whether these different personal perceptions of recovery are qualitatively different or whether they indicate varying levels along a continuum toward full recovery.

What is clear is that recovery from an ED includes a wide range of physical, symptomatic, cognitive, affective and psychosocial factors. Dawson et al. (2015) conducted a Delphi study with ED professionals, aimed at achieving a convergence of opinion about criteria for recovery from AN. They concluded that psychological and quality of life variables should be included in the definition for AN recovery, alongside weight restoration and symptom reduction. In their review of the conceptualization and operationalization of ED recovery, Bardone-Cone et al. (2018) pointed out that in recent years, there has been a general trend toward acknowledging that psychological and cognitive symptoms are important to recovery in addition to physical and behavioral indices. Nevertheless, many qualitative studies use a subjective, self-reported definition of recovery and findings can be difficult to operationalize since criteria tend to be broad and non-specific.

Various self-report instruments have been proposed to assess recovery from mental illnesses in general (Burgess et al., 2011), most of them based on the recovery model (Shanks et al., 2013). Pinto et al. (2006) proposed a self-report measure of self-efficacy to recover from an ED. Pettersen et al. (2012) proposed a 17-item “patient-related measure” of recovery from EDs containing two subscales, a psychosocial subscale and a symptom-specific subscale.

Noordenbos and Seubring (2006) compiled a 52-item checklist that included a broad range of items about symptomatic, physical, psychological, emotional, and social aspects of recovery from EDs. Fifty-seven clinicians and 41 ex-patients were asked whether or not each item on the checklist was important to recovery from EDs. There was broad agreement between the groups, although ex-patients felt more strongly than therapists that self-acceptance, a positive attitude toward the body and emotional expression were important to recovery and therapists felt more strongly than ex-patients that eating-related symptoms and physical recovery were more important (Noordenbos and Seubring, 2006). Emanuelli et al. (2012) asked 102 patients and 136 clinicians to rank the importance of these same survey items for recovery from EDs on a seven-point Likert scale. Consensus between the groups was again high, but patients ranked psychological, emotional, social and appearance-related criteria as being more important for recovery than the clinicians. An exploratory factor analysis (EFA) of this questionnaire yielded five factors: Psychological, emotional, and social criteria; weight control behaviors, non-life-threatening features, life-threatening features and evaluation of one’s own appearance.

The aim of the present study was to adapt and further explore this instrument by administering it to a sample of family members of people with EDs in addition to a sample of individuals reporting a lifetime ED diagnosis and a sample of ED clinicians. Since we wished to explore whether and to what extent opinions about the components of recovery might be associated with current levels of eating symptomatology, we administered a short measure of ED symptoms. Family members’ perception of recovery from EDs has rarely been explored in research. We also aimed to further explore the factor structure of this adapted instrument and to choose the items with the highest factor loadings to form a briefer, user-friendly multidimensional questionnaire that encompasses the main features of recovery from ED.

## Materials and Methods

### Participants

Participants in the study were 213 volunteers between the ages of 18 and 65 (mean = 32.0 ± 10.1), whom we divided into the following groups:

(1) Participants reporting a lifetime ED diagnosis (“ED group”; *n* = 118; 4 males, mean age = 28.58 + 8.8) were recruited via announcements on relevant social media sites and by word of mouth. The current (*n* = 63) or past (*n* = 32; 23 were unsure about current vs. past) diagnoses included anorexia nervosa (*n* = 60), bulimia nervosa (*n* = 31), binge eating disorder (*n* = 34), other EDs (*n* = 15). Twenty-five participants reported having experienced more than one ED. Thirty-seven of the ED participants also had a family member with a present or past ED.

(2) Eating disorder clinicians (*n* = 58; 8 males, mean age = 36.2 ± 9.1), including psychologists (*n* = 26), dietitians (*n* = 12), social workers (*n* = 10), physicians (*n* = 1), nurses (*n* = 3), expressive therapists (*n* = 3), and “others” (*n* = 15) who had worked with ED patients for at least 2 years were recruited by means of an email to the members of the Israel Association for EDs.

(3) Healthy family members of people with EDs (“healthy family members”; *n* = 37; 16 males, mean age = 36.4 + 11.6), including siblings (*n* = 17), daughters (*n* = 10), partners (*n* = 8), and mothers (*n* = 2), who reported a lifetime ED diagnosis. They were referred to the study by participants in the “ED group” and reported that they had never been diagnosed with an ED or another psychiatric disorder.

There were originally 224 participants. However, in order to minimize overlap between groups, we excluded from analyses seven therapists who reported a personal ED history, four of whom also reported having a family member with an ED, as well as four therapists who reported having a family member with an ED history but did not report a personal ED history. Data from a total of 213 participants were therefore included in analyses.

A significant between-group difference was observed for age [*F*_(2,210)_ = 17.52, *p* < 0.001]. The ED group (average age = 28.6 years, *SD* = 8.8) was younger than the ED clinicians (average age = 36.2, *SD* = 9.1) and the healthy family members (average age = 36.4, *SD* = 11.6). There was also a significant between-group difference for level of education [*F*_(2,210)_ = 35.70, *p* < 0.001], no doubt due largely to this age difference. Age was therefore entered as a covariate in the analyses relevant to the groups.

### Instruments

(1) Eating disorder cognitions and behaviors were reported by participants in all three groups using the Eating Disorder-15 (ED-15; Tatham et al., 2015). This ten-item questionnaire includes two subscales, Weight and Shape Concerns (6 items) and Eating Concerns (4 items). It has solid psychometric properties and differentiates between clinical and non-clinical populations (Tatham et al., 2015). In this study, the alpha Cronbach was 0.96.

(2) Beliefs about the components of recovery from an ED were assessed via the Eating Disorders Recovery Endorsement Questionnaire - (EDREQ), a 56-item questionnaire devised for this study. The EDREQ is based on the list of 52 recovery criteria devised by Noordenbos and Seubring (2006). Respondents were asked to indicate on a seven-point Likert scale (0 = not at all important; 6 = extremely important) to what degree they thought each criterion was important for recovery. Several adaptations were made to the original checklist. “Does not punish herself after a meal” was replaced by “Does not feel guilty after a meal” (item no. 33), since the experience of guilt after meals is very common (Burney and Irwin, 2000). One item, “monthly periods come regularly,” was considered superfluous in addition to item 18 (“has her monthly periods”) and was therefore omitted. Three items, “has no constipation,” “has no intestinal disturbances” and “has no stomach complaints” were combined into one item about digestive problems: “has no frequent digestive complaints.” Seven items were added: (1) “Weight has been stable for 3 months” (item no. 17) – A period of 3 months is often used in the context of recovery (Kordy et al., 2002). We did not exclude the item indicating stable weight for 4 weeks, however, we thought it was important to receive more specific information about the duration of weight stability so as to differentiate levels of recovery or potentially distinguish between partial and full recovery. This item was seen as being relevant not only to weight maintenance required in recovery from AN, but to an end to the weight oscillations or gain so often observed in BN and BED (Fairburn et al., 2000); (2) “Blood pressure is normal” (item no. 20) – blood pressure is often abnormal in the active phase of AN (Meczekalski et al., 2013), BN (Murialdo et al., 2007), and BED (Abraham et al., 2014); (3) “Doesn’t place others’ needs before her own” (item no. 48): Selflessness has been shown to characterize people with EDs (Bachar et al., 2002) and levels of selflessness may level off with recovery (Bachner-Melman et al., 2007); (4) “Is in contact with family members” (item no. 53) – Connection is a characteristic of recovery from an ED (Pettersen and Rosenvinge, 2002), including connection with family members (Zohar et al., 2016). This item was therefore added to the existing items “has an intimate relationship” and “has some friends”; (5) “Feels satisfied with her life,” “Feels she can trust herself,” and “Feels she can trust others in times of distress” – These three items relate to post traumatic growth following difficult life events (Tedeschi and Calhoun, 1996), which has also been connected to recovery from EDs (Moss, 2014).

Both instruments were translated into Hebrew, the native tongue of the participants, for the purposes of this study, by translation, back translation comparison and correction (Brislin, 1970).

### Procedure

This study was carried out in accordance with the recommendations of the Ethics Committee of the Ruppin Academic Center, and all subjects gave written informed consent in accordance with the Declaration of Helsinki. The protocol and informed consent procedure were approved by the Ethics Committee of the Ruppin Academic Center. Participants completed questionnaires online via Qualtrics^1^, after receiving full information about the study and providing informed consent on the first screen. Data was exported into an SPSS file and analyses were conducted using SPSS 23. All EDREQ items were entered into an EFA to identify an appropriate and meaningful factor structure. A shorter version of the questionnaire was then constructed using the highest loading items of each subscale, and the structure validity of the short version was tested using CFA. The means of the subscales were then compared between groups to identify differences in how recovery is perceived by people with personal experience of an ED, therapists, family members, and family members with personal experience of an ED. Pearson correlations were calculated between ED-15 scores and EDREQ scores for participants with personal experience of an ED, and for the whole sample, to determine whether opinions about the components of recovery are influenced by current levels of eating symptomatology.

## Results

### EFA of the EDREQ

All 56 items were entered into an EFA. Varimax rotation was used, although Promax solutions produced the identical factor structure. A four-factor solution appeared most appropriate, based on the relative slopes of the scree plot and the interpretability and conceptual clarity of the resultant factor solutions. The factors had Eigen values of 16.52, 5.78, 4.68, and 3.60, respectively (see Table 1), with a cumulative explained variance of 54.60%. Item factor loading was restricted to >0.30. Nine items loaded onto the first factor and were all connected with a lack of symptoms, so this factor was named Lack of Symptomatic Behavior (LSB). Thirteen items loaded onto the second factor and were related to body satisfaction and general self-acceptance, so this factor was named Acceptance of Self and Body (ASB). However, five of these items also loaded onto the third factor. The third factor consisted of 19 items that related to positive social interaction and emotions and was therefore named Social and Emotional Connection (AEC). The last 15 items loaded onto the fourth factor and were connected to the physical aspects of recovery, so this factor was named Physical Health (PH). Results are shown in Table 1.

**Table 1:** Exploratory factor analysis for the EDREQ (*N* = 224).

		Factor			
		
Item number	Item	LSB	ASB	SEC	PH
5	Does not take laxatives	**0.87**			
4	Does not vomit after a meal	**0.85**			
7	Does not use slimming pills	**0.84**			
6	Does not use diuretics	**0.84**			
8	Does not exercise excessively	**0.63**			
3	Does not binge	**0.58**			
2	Normal caloric intake	**0.54**			
9	Does not use too much alcohol	**0.50**			
1	Eats three meals a day	**0.44**			
11	Has a positive experience of the body		**0.81**		
12	Accepts her appearance		**0.76**		
10	Does not feel too fat		**0.71**		
31	Self-esteem is not dependent on weight		**0.70**		
30	Has adequate self-esteem		**0.70**		
14	Is not obsessed by food and weight		**0.66**		
13	Feels no need to slim excessively		**0.63**		
33	Does not feel guilty after meals		**0.59**		
54	Feels satisfied with her life		**0.44**	0.54	
37	Has a realistic image of herself		**0.41**	0.51	
36	Has no strong fear of failure		**0.39**	0.55	
35	Is not extremely perfectionistic		**0.36**	0.51	
32	Can be assertive		**0.33**	0.48	
45	Is able to handle conflicts			**0.80**	
41	Is able to handle negative emotions			**0.80**	
46	Is in touch with her own feelings			**0.79**	
42	Is able to handle positive emotions			**0.78**	
39	Is able to express her emotions in words			**0.74**	
49	Is able to make contact with others			**0.75**	
44	Dares to express a different opinion			**0.75**	
50	Is not isolated			**0.75**	
43	Is not very dependent on others’ opinions		0.32	**0.71**	
47	Doesn’t place others’ needs before her own			**0.69**	
51	Has some friends			**0.69**	
40	Is able to express her emotions non-verbally			**0.68**	
48	Participates in social activities			**0.66**	
55	Feels she can trust herself			**0.64**	
56	Feels she can trust others in times of distress			**0.64**	
53	Is in contact with family members			**0.63**	
38	Is not depressed			**0.47**	
52	Has an intimate relationship			**0.46**	
34	Can concentrate well			**0.44**	0.38
21	Body temperature is normal				**0.87**
23	Potassium values are normal				**0.80**
22	Heartbeat is normal				**0.81**
20	Endocrinological values are normal				**0.83**
24	Electrolytes are normal				**0.77**
19	Blood pressure is normal				**0.79**
26	Skin is not excessively dry				**0.73**
27	Has healthy teeth				**0.68**
25	Has no frequent digestive complaints				**0.65**
29	Is not often tired				**0.63**
28	Sleeps normally				**0.57**
18	Has her monthly periods				**0.56**
17	Weight has been stable for 3 months				**0.51**
16	Weight has been stable for 4 weeks				**0.47**
15	Weight is normal for age and height	0.40			**0.41**
Cronbach’s alpha: 0.95 (total)		0.85	0.90	0.95	0.93


*Only factor loadings of over 0.30 are shown. LSB, Lack of Symptomatic Behavior; ASB, Acceptance of Self and Body; SEC, Social and Emotional Connection; PH, Physical Health*. Bold values indicates the subscale in which the item was included.

In an attempt to shorten the questionnaire and to eliminate items that loaded onto more than one factor, a shorter version of the questionnaire was constructed with only the seven highest loading items of each subscale. A Varimax rotation was used in a further EFA and restricted to a four-factor solution (see Table 2). The factors had Eigen values of 7.57, 4.22, 4.09, and 2.78, respectively, with a cumulative explained variance of 66.64%. Item factor loading was restricted to >0.30 and seven items loaded onto each factor.

**Table 2:** Exploratory factor analysis for the EDREQ short version (*N* = 224).

Item number	Item	Factor			
		
		LSB	ASB	SEC	PH
21	Body temperature is normal				0.92
23	Potassium values are normal				0.88
22	Heartbeat is normal				0.88
20	Endocrinological values are normal				0.86
24	Electrolytes are normal				0.86
19	Blood pressure is normal				0.82
26	Skin is not excessively dry				0.72
41	Is able to handle negative emotions			0.85	
46	Is in touch with her own feelings			0.84	
45	Is able to handle conflicts			0.84	
42	Is able to handle positive emotions			0.83	
39	Is able to express her emotions in words			0.77	
44	Dares to express a different opinion			0.76	
49	Is able to make contact with others			0.69	
5	Does not take laxatives	0.93			
4	Does not vomit after a meal	0.90			
6	Does not use diuretics	0.90			
7	Does not use slimming pills	0.88			
8	Does not exercise excessively	0.63			
3	Does not binge	0.60			
2	Normal caloric intake	0.46			
11	Has a positive experience of the body		0.88		
12	Accepts her appearance		0.85		
10	Does not feel too fat		0.79		
14	Is not obsessed by food and weight		0.72		
13	Feels no need to slim excessively		0.70		
30	Has adequate self-esteem		0.64		
31	Self-esteem is not dependent on weight		0.64		
	Chronbach’s alpha	0.87	0.88	0.92	0.94


*LSB, Lack of Symptomatic Behavior; ASB, Acceptance of Self and Body; SEC, Social and Emotional Connection; PH, Physical Health*.

### Confirmatory Factor Analysis of the EDREQ

**FIGURE 1:**
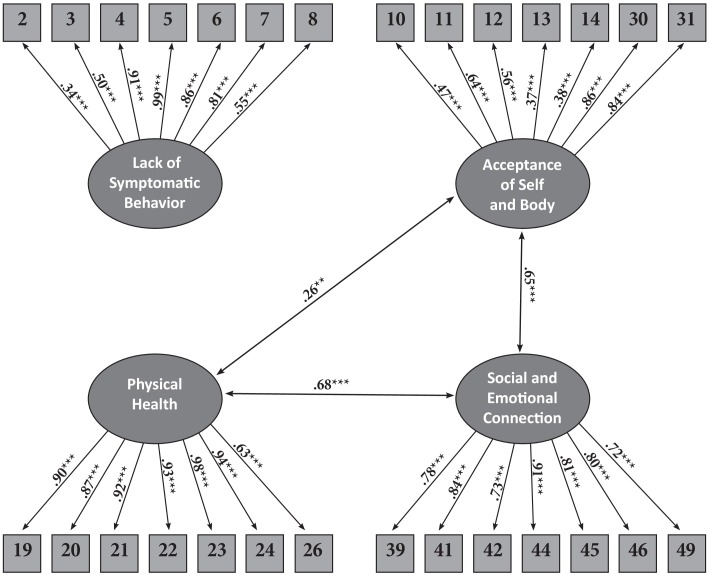
Confirmatory factor analysis for the short version of the EDREQ.

Confirmatory factor analysis (CFA) examines the consistency of constructs that are theorized to have a specific structure. The hypothesized structure is entered to constrain the analysis, after which the analysis examines the fit of the actual data to this hypothesized model. To confirm the hypothesized structure, the CFA needs to show good model-fit-indices, and if these are inadequate, the hypothesized model is rejected. We used CFA to test the structure validity of the shortened recovery questionnaire, and chose the following values for our model to be accepted: CFI (Comparative Fit Index) > 0.90 (Bentler and Bonett, 1980), and RMSEA (root mean square error of approximation) < 0.08 (Browne and Cudeck, 1993; see Figure 1). The Chi-square goodness-of-fit index presented an excellent fit for the data, χ^2^(314, *N* = 224) = 493.63, *p* > 0.001; CFI = 0.96; RMSEA = 0.05; standardized RMR = 0.06.

Intercorrelations of the EDREQ subscales were examined and are presented in Table 3. SEC was positively correlated with PH and with ASB. PH had a significant but small positive correlation with both ASB and LSB and SEC had a significant but small positive correlation with LSB.

**Table 3:** Inter-correlations of the EDREQ subscale-scores (*N* = 224).

	PH	SEC	ASB	LSB
PH		0.34^∗∗∗^	0.13^∗^	0.13^∗^
SEC			0.33^∗∗∗^	0.13^∗^
ASB				0.07
Mean (*SD*)	4.69 (1.45)	5.19 (1.28)	5.58 (1.09)	6.20 (0.98)


*LSB, Lack of Symptomatic Behavior; ASB, Acceptance of Self and Body; SEC, Social and Emotional Connection; PH, Physical Health.*^∗^p < 0.05, ^∗∗^p < 0.01, ^∗∗∗^p < 0.001 (2-tailed).

**FIGURE 2:**
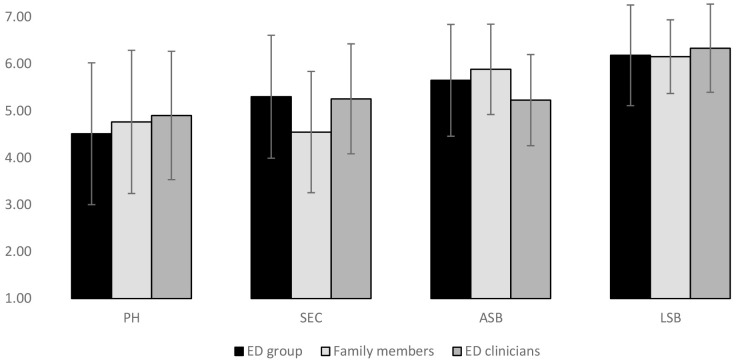
Differences between groups on perceived importance of recovery subscales. LSB, Lack of Symptomatic Behavior; ASB, Acceptance of Self and Body; SEC, Social and Emotional Connection; PH, Physical Health.

Lack of Symptomatic Behavior was rated by all groups as the most salient component of recovery. Overall, ASB was next in line, followed by SEC and PH in that order. However, a MANOVA test revealed significant differences between the groups (ED group, healthy family members and ED therapists with age held constant) in the importance they attributed to each of the recovery subscales [*F*_(8,414)_ = 4.00, *p* < 0.001]. These differences can be seen in Figure 2. Groups differed statistically on SEC [*F*_(2,209)_ = 6.13, *p* = 0.003] with the ED group and ED therapists rating this as a highly important facet of recovery, and family members rating it as less central. *Post hoc* tests revealed that healthy family members differed significantly from both the ED group (*p* = 0.008) and from therapists (*p* = 0.03) on their ranking of the SEC items. The groups also differed statistically on ABS [*F*_(2,209)_ = 4.74, *p* = 0.01] with healthy family members rating self-acceptance as more important than people with an ED history and therapists. *Post hoc* tests revealed a significant statistical difference between healthy family members and ED therapists on the ranking of the ABS (*p* = 0.02).

For the whole sample, none of the correlations between ED-15 scores and the subscales of the EDREQ were significant. For the ED group, only one correlation was significant, between ED-15 scores and PH (*r* = 0.20, *p* = 0.04).

## Discussion

In this paper we have presented the EDREQ, a further elaboration and refinement of the work based on a recovery checklist proposed by Noordenbos and Seubring (2006) and developed by Emanuelli et al. (2012). While there is still no consensus definition of recovery from an ED (Bardone-Cone et al., 2018), we present a psychometrically sound questionnaire containing items that people with a personal ED history, their family members and therapists all define as important components of recovery. The EDREQ includes four subscales, the Absence of Symptomatic Behavior, Acceptance of Self and Body, Social and Emotional Connection, and Physical Health. When the seven highest loading items from each of the four factors were selected from the original 56 items, the internal reliability of the scales and their structural validity remained excellent, resulting in a user-friendly, 28-item scale that makes it possible to measure recovery at any point in time as a continuous variable. The EDREQ is appropriate for use in both clinical and research settings, and responses can be noted via self-report or adapted for report by a clinician.

In terms of the medical model, the EDREQ assesses recovery both in terms of a lack of clinical symptoms of ED and in terms of biological health, as has been previously proposed for definitions of recovery (Couturier and Lock, 2006). People with a personal history of ED, family members of people with ED and therapists who treat ED all ranked a Lack of Symptomatic Behavior as being the most salient component of recovery from an ED, and there were no significant group differences for the rating of the importance of this subscale. All parties therefore confirmed the basic idea and backbone of the medical model that recovery is, first and foremost, an improvement in symptomatic behavior. Physical Health, including normal body temperature, heart rate, body weight, and electrolyte levels, was attributed lower importance than all three other subscales by people with a personal history of ED and ED clinicians, but not by family members, who ranked Social and Emotional Connection as less important. However, as with a Lack of Symptomatic Behavior, there were no significant group differences in the attributed level of importance of Physical Health, although within the ED group, participants with more severe ED pathology tended to stress the importance of PH in recovery more than participants who were less symptomatic. Overall, it seems fair to say that there was broad agreement between patients, family members and therapists about the relative importance of the features of the medical model of recovery from EDs.

Beyond medical and symptomatic factors, the EDREQ incorporates two emotional and psychosocial facets of psychosocial health: Acceptance of Self and Body, and Social and Emotional Connection, which includes a healthy dose of self-assertion (“able to express her emotions in words,” “dares to express a different opinion”). Traditionally peripheral in clinical practice and research on recovery, these concepts, with the possible exception of body acceptance, are more global and less specifically focused on ED symptomatology than the other two subscales. Yet previous research has clearly shown that emotional (Federici and Kaplan, 2008), psychological (Jones et al., 2005) and social factors (Linville et al., 2012) should be considered important elements of recovery. There were some significant differences in the importance attributed to these subscales by the different groups. Family members of people with EDs rated Social and Emotional Connection lower than the other groups and the Acceptance of Self and Body higher than ED therapists. The perspective of healthy family members on recovery from an ED may therefore differ somewhat from that of patients and clinicians. Nevertheless, the statistically significant group differences observed were not very dramatic and did not form a clear or interpretable pattern. Emanueli et al. (2012) similarly found that patients and therapists rated the importance of the various components of recovery differently for only two of the five factors they extracted, and the differences were not very large. Vanderlinden et al. (2007) also found that therapists and patients share a similar view about necessary components of the recovery process. It therefore seems fair to conclude that despite the absence of a standardized definition of recovery from ED, there is a general consensus about its components.

We concede that it is by no way a perfect solution to use a numerical score at one specific point of time to capture an ongoing process such as recovery. Recovery from an ED is a long, continuous, non-linear process (Lindgren et al., 2015) that changes during treatment and over time (Garrett, 1997; Lamoureux and Bottorff, 2005; Shohet, 2007; Leslie, 2014), yet in fact increases linearly in the long term with duration of follow-up (Steinhausen, 2009; Eddy et al., 2017). Nevertheless, periodical assessment of recovery levels during therapy and follow-up using the EDREQ as a clinical tool could provide regular and relevant clinical feedback to patients, therapists and family members indicative of the recovery trajectory. One-point recovery scores could provide a quantitative measure of recovery from ED for use in studies of outcome, treatment efficacy, metanalytic studies, multi-site comparisons and other recovery-related research.

This study has several limitations. First, the sample is relatively small and the ED and clinician groups were overwhelmingly female, so the endorsement of EDREQ items may not be generalizable to males and other samples of people with ED, family members and clinicians. The results of this study therefore require replication with different and larger samples. Second, the EDREQ was built from a pre-determined set of items, so that other criteria may have been endorsed if included. For example, Garrett (1997) stressed spirituality as being central in recovery and no items addressed this. Third, a self-report questionnaire would not be adequate in terms of determining current ED diagnosis, for which additional clinical data is needed. Finally, in this study the EDREQ was administered to seek endorsement of items indicative of recovery and was not examined as a clinical measure of recovery levels.

It is therefore essential to follow up this and other studies on recovery from ED and its assessment with further research. The EDREQ should be examined beyond theoretical endorsement of its items as a clinical tool for people with ED, their therapists and family members. Such a clinical tool could be refined and used alone or in conjunction with other criteria as a measure of recovery from ED in clinical work and research. In parallel, to improve treatment and increase the reliability of outcome studies, a standardized definition of recovery from ED should be pursued, via research, conference symposia, ED organization task forces and consensus statements. In the context of clinical implications, future research should also examine changes in the EDREQ subscale scores over time during the process of recovery to test the hypotheses that symptomatic and physical improvement precede the more psychosocial and emotional expressions of recovery (Fennig et al., 2002), and that a positive attitude toward the body is one of the most difficult aspects of recovery to achieve (Beresin et al., 1989). Social and Emotional Connection subscale scores should be examined in relation to recovery from disorders other than EDs and similarities and differences between recovery from different disorders investigated. Another subject for future research is gender differences, since these appear to exist in recovery from ED. Males, for example, tend to emphasize more than females the importance of relapse prevention strategies (Björk and Ahlstrom, 2008) and curbing compulsive physical training (Björk et al., 2012), whereas females tend to place more emphasis on emotional coping and problem solving (Björk et al., 2012). The relationship between levels of recovery from EDs and levels of symptomatology also seems worthy of investigation. Do recovery levels increase as a direct function of decreasing symptomatology, or can psychosocial and emotional recovery progress despite ongoing symptoms and physical problems, supporting the recovery model?

Other terms that need to be defined and differentiated from recovery include remission, relapse and reoccurrence (Frank et al., 1991; Ackard et al., 2014). Khalsa et al. (2017) reviewed the literature on relapse, recovery and remission from EDs, and propose a set of standardized criteria for relapse, recovery and remission. For example, it needs to be determined whether remission differs from recovery only in terms of duration, as suggested by Kordy et al. (2002) or also in terms of the severity of symptomatology, as suggested by Khalsa et al. (2017). The EDREQ should also be examined in relation to these concepts and definitions. Since some studies have proposed different criteria for recovery from AN subtypes and from BN (Kordy et al., 2002), it should also be explored whether slightly different versions of the EDREQ would more accurately tap the nuances between recovery from AN, BN, BED, and other EDs. Ideally, reliable and standardized cutoff points for partial and full recovery would be sought and validated in long-term, prospective studies using a clinical version of this questionnaire.

We propose the 28-item EDREQ as a psychometrically sound foundation for a self-report or clinician-reported instrument for use by people with EDs, their family members and therapists, and researchers to assess and monitor patients’ progress in overcoming an ED. It could be administered at one time point, or at several time-points along an individual’s trajectory between profound illness and health. Clinically, such a questionnaire stands to assist in setting and refining therapeutic goals throughout therapy, and in establishing standardized, comparable norms for recovery levels in research. The inclusion of emotional and psychosocial aspects of recovery in addition to symptomatic and medical aspects is important to expand treatment goals and the concept of recovery from EDs beyond symptom relief and the absence of disease markers.

## Data Availability

The datasets generated for this study are available on request to the corresponding author.

## Author Contributions

RB-M conceived and designed the study, supervised data collection, and wrote the bulk of the paper. LL-A conducted all statistical analyses, wrote the “Results” section, and contributed to the rest of the text. AZ contributed significantly to data analysis and writing of the manuscript. SL collected the data and assisted with data analysis, writing and preparation of the manuscript for submission.

## Conflict of Interest Statement

The authors declare that the research was conducted in the absence of any commercial or financial relationships that could be construed as a potential conflict of interest.

## Footnotes

1 www.qualtrics.com
